# The case for a Canadian standard for 2SLGBTQIA+ medical education

**DOI:** 10.1503/cmaj.202642

**Published:** 2021-04-19

**Authors:** Miranda Schreiber, Tehmina Ahmad, Michael Scott, Kevin Imrie, Saleem Razack

**Affiliations:** University of Toronto (Schreiber); Divisions of Endocrinology & Metabolism (Ahmad) and Hematology (Scott, Imrie), Department of Medicine, and Odette Cancer Centre (Imrie), and Sunnybrook Health Sciences Centre (Imrie), University of Toronto, Ont.; Department of Pediatrics and Institute for Health Sciences Education (Razack), McGill University, Montréal, Que.

**KEY POINTS**
- Two-spirit, lesbian, gay, bisexual, transgender, queer, intersex and asexual (2SLGBTQIA+) Canadians experience disproportionately poor health outcomes.
- Intersecting oppressive systems, such as white supremacy and colonialism, create worsened health outcomes for 2SLGBTQIA+ people who are Black or Indigenous or both.
- Health care providers receive inadequate training in 2SLGBTQIA+ care, contributing to suboptimal experiences for 2SLGBTQIA+ patients.
- Integrating 2SLGBTQIA+ content into medical curricula has inherent challenges but has been shown to be an effective step to improving competence.
- National standardization mandating inclusion of 2SLGBTQIA+ curricula within undergraduate and postgraduate medical education will better support health care for this community.

Two-spirit, lesbian, gay, bisexual, transgender, queer, intersex and asexual individuals (2SLGBTQIA+) in Canada experience worse health outcomes than their heterosexual, cisgender peers. The historical and ongoing structural oppression of 2SLGBTQIA+ people, to which the health care system contributes, drives these disparities. In the 19th century, Western medicine classified homosexuality as a medical condition. The *Diagnostic and Statistical Manual of Mental Disorders* (DSM) explicitly pathologized homosexuality until the early 1970s, and continues to characterize gender dysphoria as a mental health diagnosis. Today, negative encounters with physicians lead 2SLGBTQIA+ people to avoid seeking health care, and many physicians report feeling underprepared in treating 2SLGBTQIA+ patients. Although medical educators in Canada and the United States have acknowledged the need to prepare trainees to provide 2SLGBTQIA+ patients with informed, compassionate care,^1^ medical education in Canada related to health of 2SLGBTQIA+ patients remains sparse and inconsistent. We consider the promise and challenges of integrating 2SLGBTQIA+ content into medical education across Canadian medical schools. We argue for the creation of a national standard for 2SLGBTQIA+ health care education and physician competency objectives in Canada.

## What affects the health of 2SLGBTQIA+ patients in Canada?

The 2019 House of Commons Report on the health of LGBTQIA2 communities concluded that LGBTQIA2 people in Canada experience worse health outcomes than their cisgender, heterosexual counterparts,^2^ including disproportionate rates of cancer, chronic fatigue and heart disease.^2^ The report found that 2SLGBTQIA+ people are less likely to have a family doctor, and are more likely to live with chronic health conditions, poor mental health and substance-dependence disorders.^2^,^3^

The historical and ongoing structural oppression of 2SLGBTQIA+ people has produced these health disparities. The pathologization of homosexuality in the 19th century led Western physicians to subject 2SLGBTQIA+ people to lobotomies, electric shock treatments and chemical castrations until the 1970s.^4^ Homosexuality was classified as a mental disorder in the DSM until 1973 and, the current edition (published in 2013) includes “gender dysphoria” as a mental disorder, pathologizing transgender individuals.^4^,^5^ The AIDS crisis, which surged in the 1980s, was met with governmental negligence on both sides of the US–Canada border. The Reagan administration was reluctant to fund AIDS research, taking until 1985 to even acknowledge the AIDS epidemic.^6^ Although elected in 1984, then–Canadian Prime Minister Brian Mulroney did not acknowledge the existence of AIDS until 1989, and 2SLGBTQIA+ community groups and Canadian researchers had to work to provide people with experimental medications without government support.^7^,^8^

Structural injustices continue to sustain the 2SLGBTQIA+ health gap in 2021. 2SLGBTQIA+ people are disproportionately unhoused, with 2SLGBTQIA+ youth making up 5% of youth but 40% of unhoused youth in Toronto.^9^,^10^ As one of the principal determinants of health is access to shelter, the ongoing housing crisis disproportionately harms 2SLGBTQIA+ people.^9^,^10^ Furthermore, because 2SLGBTQIA+ people — especially those who are Black or Indigenous or both — are overrepresented in low-wage sectors, insufficient institutional support for those living in poverty also contributes to their suboptimal health outcomes.^11^

The medical system itself also perpetuates the 2SLGBTQIA+ health gap. National- and state-level surveys in the US have shown that negative clinical experiences drive 2SLGBTQIA+ people to avoid seeking health care, and a 2011 focus group study reported that 2SLGBTQIA+ patients feel pressure to educate their own health care professionals.^12^,^13^ According to a 2017 survey of transgender individuals in Ontario, 43% had had an unmet health need in the past year.^14^ Another 2017 survey of 45 practising clinicians found consistent knowledge gaps about 2SLGBTQIA+ health; a literature review of existing health-related risk factors for 2SLGBTQIA+ people found that clinicians overlook these variables in patient interviews.^15^,^16^ The inability to provide sufficient evidence-based assessments has led to incorrect advice from clinicians, which increased the risk of cervical cancer for members of the 2SLGBTQIA+ community.^17^

Intersectional analysis^18^ shows that in Canada, a plurality of oppressive systems produce unique challenges for 2SLGBTQIA+ people harmed by multiple systems. Two-Spirit People’s barriers to health care are compounded by colonialism, racism and cultural insensitivity, along with homophobia and heterosexism.^19^–^21^ Community-based qualitative research shows that gay Black men in Canada also encounter unique and substantial barriers to health care,^22^ and 2SLGBTQIA+ people who migrate from Africa and the Caribbean experience disproportionate trauma and mental illness.^23^ As the Canadian Parliamentary Committee report concluded, “each subgroup within LGBTQIA2 communities has specific health vulnerabilities [and] … intersectional analysis is needed.”^2^

## How do medical schools in the US and Canada currently educate students about 2SLGBTQIA+ health?

Medical education about 2SLGBTQIA+ health is currently limited and inconsistent in Canada and the US.^24^ It has failed to develop and formalize content related to the health of 2SLGBTQIA+ patients, while simultaneously perpetuating informal bias and misinformation about 2SLGBTQIA+ communities. A 1991 survey of all US medical schools reported a national average of 3 hours and 26 minutes spent studying the health of homosexual patients.^25^ Twenty years later, a survey of North American medical schools found the median number of hours spent teaching LGBT-related content over 4 years was only 5 hours; medical trainees lacked clinical exposure, and fewer than 35% of medical schools surveyed studied the health of transgender patients.^24^ A 2016 national survey found that, although 95% of Canadian medical students agreed that health care specific to transgender patients was important, fewer than 10% felt they were sufficiently knowledgeable to provide it.^26^

Deficiencies in 2SLGBTQIA+ undergraduate medical education have downstream effects on physician attitudes and competence. A 2019 multicentre online evaluation of the preparedness of residents to treat 2SLGBTQIA+ patients found that trainees of all levels were insufficiently prepared with regard to terminology, knowledge of preventive care, mental health care and sexual health.^27^ The evaluation found that trainees did not know that health disparities specific to this community existed.^27^ Emergency medicine training programs provide an average of only 45 minutes of instruction specific to LGBT patients.^28^ Finally, 80% of practising endocrinologists have not received training in care of transgender individuals, despite 80% of survey participants stating they have cared for transgender patients.^29^

The Association of American Medical Colleges argued in 2015 that the absence of medical education related to 2SLGBTQIA+ patients, along with conservative institutional climates, produces a hidden heteronormative and cisnormative curriculum that implies the health of 2SLGBTQIA+ patients is unimportant.^1^ A 2017 survey of medical students at the University of Ottawa found 41% had witnessed anti-LGBT behaviour from peers or professors, or both.^30^ Furthermore, medical examination questions perpetuate stigma by continually associating 2SLGBTQIA+ patient scenarios with HIV and sexually transmitted diseases.^31^

The Association of American Medical Colleges has indicated the need for medical institutions to “prepare students to respond effectively, compassionately, and professionally to the needs of all types of patients … regardless of patients’ sexual orientation or gender identity.”^32^ The Association of American Medical Colleges furthermore has identified the “[l]ack of LGBT-content” on national examinations by accreditation bodies as a significant weakness in educational standards.^32^

## What are the barriers to developing a curriculum that offers adequate 2SLGBTQIA+ medical education?

The lack of health data specific to 2SLGBTQIA+ people complicates development of 2SLGBTQIA+ curricula in medicine. The Canadian Census will first collect multidimensional 2SLGBTQIA+ data in May 2021, and the exact number of 2SLGBTQIA+ people in Canada is not known with certitude.^2^ Moreover, little is known about which methodologies best serve students and 2SLGBTQIA+ patients, as few curricular strategies have undergone evaluation. 33 Limited distribution of comprehensive resources for 2SLGBTQIA+ medical students and resident trainees also makes collaboration and research challenging, especially as there are few opportunities for 2SLGBTQIA+ health experts to work with medical trainees.^1^

## Can better medical education improve health outcomes for 2SLGBTQIA+ people in Canada?

As Sawning and colleagues wrote, “lack of provider education is only one small piece” of the 2SLGBTQIA+ health gap; interventions in medical education are not a cure-all.^34^,^35^ Development of 2SLGBTQIA+ curricula in medical education has inherent limitations, as it leaves intact the oppressive structures that ultimately produce the 2SLGBTQIA+ health gap, such as white supremacy, colonialism and the housing crisis.^36^

Nevertheless, experts, community members, researchers and learners recommend integrating 2SLGBTQIA+ content into medical curricula as a strategy to reduce suboptimal health outcomes. The House of Commons Committee, in its recommendations for action to reduce the gap in health care for 2SLGBTQIA+ people, proposed improving education about the health needs of 2SLGBTQIA+ people for all health care providers.^2^ A survey of Canadian medical students ultimately suggested fundamental curricular changes as a way to increase literacy about health care specific to transgender patients.^26^ Increasingly, literature recommends educating health care practitioners according to a pre-established standard about intersecting community needs, to support refugees and migrants who are 2SLGBTQIA+ and women who are HIV positive.^23^,^35^

Furthermore, the integration of information about 2SLGBTQIA+ health into medical curricula has been shown to improve both 2SLGBTQIA+ health literacy among physicians and clinical experiences for 2SLGBTQIA+ people. Studies of 2SLGBTQIA+ workshops at the Northern Ontario School of Medicine and the University of Louisiana found that such interventions were effective and informative and revealed a gap in learners’ knowledge; the studies concluded that medical education can play a role in ending health disparities. 33,35 Even a few hours of additional exposure to 2SLGBTQIA+ education increased the confidence of learners in Canada and the US to treat 2SLGBTQIA+ patients.^33^,^37^ Greater exposure to 2SLGBTQIA+ medical education has been correlated with improved sexual history-taking, more comprehensive preventive health counselling and fewer signs of prejudice during patient intake interviews.^33^ Therefore, the inclusion of 2SLGBTQIA+ content in medical curricula is an incomplete, but substantive, intervention on behalf of 2SLGBTQIA+ people.

## How could 2SLGBTQIA+ content be implemented in undergraduate and postgraduate medical curricula?

As of the time of writing, bodies such as the Association of Faculties of Medicine of Canada, the Royal College of Physicians and Surgeons of Canada, and the College of Family Physicians Canada have no explicit assessment objectives mandating that graduating residents and medical trainees must show knowledge or management skills specifically related to health care for 2SLGBTQIA+ patients. Although Canadian medical schools belong to the Association of American Medical Colleges and can access its resources, there are no Canadian regulatory bodies mandating students and clinicians demonstrate competence in providing health care to 2SLGBTQIA+ patients. The Association of American Medical Colleges developed a list of LGBTQ health competencies, but we contend that relying on guidelines designed for the health care system and needs of the US is inadequate for 2SLGBTQIA+ people in Canada, especially those who are Black or Indigenous or both.

The shift in knowledge acquisition over the last decade supports the need for changes in medical training programs. Competency-based medical education presents an opportunity to begin integrating the medical needs of 2SLGBTQIA+ populations into clinical assessment and care.^34^,^38^,^39^ These changes could be implemented in 3 phases.

To improve clinician literacy about health care for 2SLGBTQIA+ patients, medical education can better integrate 2SLGBTQIA+ content into curricula using case-based learning, standardized patient interviews and longitudinal didactic lectures throughout undergraduate and postgraduate training.^1^ This integration should be carried out in partnership with 2SLGBTQIA+ community organizations and patients and should attend to the systemic barriers to health care that racialized patients experience, especially those who are Black or Indigenous or both.^23^,^39^ Health disparities must be traced to the oppressive structures that produce them.^40^ Lectures on issues specific to 2SLGBTQIA+ patients could discuss terminology, history, manoeuvres for physical examinations, hormone therapy assessments, safe sexual practices, fertility concerns, cancer screening, mental health and substance use concerns. This integration should not be purely additive; showing that health issues in 2SLGBTQIA+ people manifest in *all* disciplines of medicine is important.^41^ Furthermore, providing resources for 2SLGBTQIA+ trainees, staff, patients and physicians to collaborate on curricular design, outreach or research projects would proliferate knowledge about the health of 2SLGBTQIA+ patients.

To improve clinical encounters for 2SLGBTQIA+ people, programs could train learners to better interact with 2SLGBTQIA+ patients during standardized patient interviews and clinical rotations. Case-based clinical scenarios would be trauma informed and provide training on avoiding assumptions about identity, as well as on medical racism, mental health, medical trauma, sexual health and addiction. Cases would capture the varying needs of different age demographics and include intersectional analysis; scenarios should also include how to direct patients to relevant community resources and support.^34^,^39^–^41^ Medical schools and teaching sites should also be supportive environments for 2SLGBTQIA+ students and staff, so that students learn both formally and informally to provide dignified care.^1^

Finally, to enact structural change and more broadly target the sources of the health gap for 2SLGBTQIA+ people, medical institutions must consider how they as elite institutions sustain classism, homophobia, transphobia, white supremacy and colonialism; this reflexive process also should consider the historical role of the medical institution as an instrument of such oppression. This structural reform requires an audit of existing curricular materials for outdated content in partnership with community organizations. Health literacy related to 2SLGBTQIA+ people should be incorporated into all medical fields, making adjustments to existing curricula to include the needs of 2SLGBTQIA+ patients.^1^ Ultimately, any national “standard” should ensure that educational materials suit localized needs of 2SLGBTQIA+ people. An enviroscan of each medical institution should be performed to better understand local educational needs and accessibility concerns for 2SLGBTQIA+ patients and students.

## Conclusion

Currently, no standardized set of proficiencies exist in Canada to address the health of 2SLGBTQIA+ patients. Although Canadian institutions have shown progressive changes, no guideline or resource exists to lead curricular transformations that aim to improve the health of 2SLGBTQIA+ patients in Canada. We call on the Association of Faculties of Medicine of Canada, Royal College of Physicians and Surgeons of Canada and College of Family Physicians of Canada to create competency-based and licensure-related standards that hold programs accountable for providing this training so that the health care needs of 2SLGBTQIA+ people living in Canada may be better served.
